# RETRACTED: Wigner et al. The Impact of Chronic Mild Stress and Agomelatine Treatment on the Expression Level and Methylation Status of Genes Involved in Tryptophan Catabolic Pathway in PBMCs and Brain Structures. *Genes* 2020, *11*, 1093

**DOI:** 10.3390/genes17080972

**Published:** 2026-08-19

**Authors:** Paulina Wigner, Ewelina Synowiec, Paweł Jóźwiak, Piotr Czarny, Katarzyna Białek, Michal Bijak, Janusz Szemraj, Piotr Gruca, Mariusz Papp, Tomasz Sliwinski

**Affiliations:** 1 Laboratory of Medical Genetics, Faculty of Biology and Environmental Protection, University of Lodz, 90-236 Lodz, Poland; paulina.wigner@gmail.com (P.W.); ewelina.synowiec@biol.uni.lodz.pl (E.S.); biaalek.k@gmail.com (K.B.); 2 Department of Cytobiochemistry, Faculty of Biology and Environmental Protection, University of Lodz, 90-236 Lodz, Poland; pawel.jozwiak@biol.uni.lodz.pl; 3 Department of Medical Biochemistry, Medical University of Lodz, 92-216 Lodz, Poland; piotr.czarny@umed.lodz.pl (P.C.); janusz.szemraj@umed.lodz.pl (J.S.); 4 Biohazard Prevention Centre, Faculty of Biology and Environmental Protection, University of Lodz, 90-236 Lodz, Poland; michal.bijak@biol.uni.lodz.pl; 5 Institute of Pharmacology, Polish Academy of Sciences, 31-343 Krakow, Poland; gruca@if-pan.krakow.pl (P.G.); nfpapp@cyfronet.pl (M.P.)

The journal retracts the article titled, “The Impact of Chronic Mild Stress and Agomelatine Treatment on the Expression Level and Methylation Status of Genes Involved in Tryptophan Catabolic Pathway in PBMCs and Brain Structures” [1], cited above.

Following publication, concerns were brought to the attention of the Editorial Office regarding the integrity of images presented in this article [1].

In accordance with the journal’s standard procedures, the Editorial Office and Editorial Board conducted an investigation that identified indications of inappropriate image editing and duplication within Figure 5. Although the authors cooperated with the investigation, the explanations and supporting materials provided were not deemed sufficient by the Editorial Board to resolve the identified concerns. Consequently, the Editorial Board has lost confidence in the reliability of the reported findings and has decided to retract this publication.

This retraction was approved by the Editor-in-Chief of the journal Genes.

The authors have been informed of this retraction and have indicated their disagreement with this decision.
